# Evaluation of the Serologic Cross-Reactivity between Transmissible Gastroenteritis Coronavirus and Porcine Respiratory Coronavirus Using Commercial Blocking Enzyme-Linked Immunosorbent Assay Kits

**DOI:** 10.1128/mSphere.00017-19

**Published:** 2019-03-13

**Authors:** Ronaldo Magtoto, Korakrit Poonsuk, David Baum, Jianqiang Zhang, Qi Chen, Ju Ji, Pablo Piñeyro, Jeffrey Zimmerman, Luis G. Giménez-Lirola

**Affiliations:** a College of Veterinary Medicine, Iowa State University, Ames, Iowa, USA; b College of Liberal Arts and Sciences, Iowa State University, Ames, Iowa, USA; UMKC School of Medicine

**Keywords:** ELISA, transmissible gastroenteritis virus, antibody, cross-reactivity, porcine respiratory coronavirus, serum, swine

## Abstract

This study compared the performances of three commercial transmissible gastroenteritis virus/porcine respiratory coronavirus (TGEV/PRCV) blocking enzyme-linked immunosorbent assays (ELISAs) using serum samples (*n* = 528) collected over a 49-day observation period from pigs inoculated with TGEV strain Purdue (*n* = 12), TGEV strain Miller (*n* = 12), PRCV (*n* = 12), or with virus-free culture medium (*n* = 12). ELISA results were evaluated both with “suspect” results interpreted as positive and then as negative. All commercial kits showed excellent diagnostic specificity (99 to 100%) when testing samples from pigs inoculated with virus-free culture medium. However, analyses revealed differences between the kits in diagnostic sensitivity (percent TGEV- or PRCV-seropositive pigs), and all kits showed significant (*P < *0.05) cross-reactivity between TGEV and PRCV serum antibodies, particularly during early stages of the infections. Serologic cross-reactivity between TGEV and PRCV seemed to be TGEV strain dependent, with a higher percentage of PRCV-false-positive results for pigs inoculated with TGEV Purdue than for TGEV Miller. Moreover, the overall proportion of false positives was higher when suspect results were interpreted as positive, regardless of the ELISA kit evaluated.

**IMPORTANCE** Current measures to prevent TGEV from entering a naive herd include quarantine and testing for TGEV-seronegative animals. However, TGEV serology is complicated due to the cross-reactivity with PRCV, which circulates subclinically in most swine herds worldwide. Conventional serological tests cannot distinguish between TGEV and PRCV antibodies; however, blocking ELISAs using antigen containing a large deletion in the amino terminus of the PRCV S protein permit differentiation of PRCV and TGEV antibodies. Several commercial TGEV/PRCV blocking ELISAs are available, but performance comparisons have not been reported in recent research. This study demonstrates that the serologic cross-reactivity between TGEV and PRCV affects the accuracy of commercial blocking ELISAs. Individual test results must be interpreted with caution, particularly in the event of suspect results. Therefore, commercial TGEV/PRCV blocking ELISAs should only be applied on a herd basis.

## Abstract (precis)

Current measures to prevent TGEV from entering a naive herd include quarantine and testing for TGEV-seronegative animals. However, TGEV serology is complicated due to the cross-reactivity with PRCV, which circulates subclinically in most swine herds worldwide. Conventional serological tests cannot distinguish between TGEV and PRCV antibodies; however, blocking ELISAs using antigen containing a large deletion in the amino terminus of the PRCV S protein permit differentiation of PRCV and TGEV antibodies. Several commercial TGEV/PRCV blocking ELISAs are available, but performance comparisons have not been reported in recent research. This study demonstrates that the serologic cross-reactivity between TGEV and PRCV affects the accuracy of commercial blocking ELISAs. Individual test results must be interpreted with caution, particularly in the event of suspect results. Therefore, commercial TGEV/PRCV blocking ELISAs should only be applied on a herd basis.

## INTRODUCTION

Transmissible gastroenteritis virus (TGEV) and porcine respiratory coronavirus (PRCV) are enveloped single-stranded positive-sense RNA viruses belonging to the Alphacoronavirus 1 species within the genus Alphacoronavirus in the family *Coronaviridae*. Transmissible gastroenteritis virus is a highly contagious virus that causes enteric disease characterized by vomiting, severe diarrhea, and high mortality in piglets in TGEV/PRCV-naive herds. The virus was first described by Doyle and Hutchings in 1946 in the United States and subsequently reported worldwide (1–3). Porcine respiratory coronavirus is a naturally occurring spike gene deletion (170 to 190 kDa) mutant of TGEV first isolated in Belgium in 1984 (4). It infects the upper respiratory tract, tonsils, or lungs, with limited intestinal replication (4, 5). Porcine respiratory coronavirus itself does not appear to be an important primary pathogen, with the exception of its contribution to the porcine respiratory disease complex (6).

TGEV and PRCV share biological and molecular features but differ in their epidemiology, clinical presentation, and pathogenesis. Real-time reverse transcription-PCR (rRT-PCR) and multiplex microarray hybridization using primers targeting the 5′ region of the S gene spanning the deletion region in PCRV strain are commonly used for the diagnosis of TGEV and differentiation of TGEV and PRCV (7–10). Serum antibodies provide serological evidence of TGEV or PRCV infection, but PRCV-infected pigs produce antibodies that cross-react and cross-neutralize TGEV, i.e., conventional serological tests cannot differentiate between TGEV- and PRCV-infected animals. This presents a complication for TGEV seroprevalence studies and serological surveys of sows or slaughterhouse swine tested for international trade (5).

To address the issue of cross-reactivity, monoclonal antibodies targeting antigenic regions of TGEV that have been deleted from the PRCV S protein (11–17) have been used to develop blocking enzyme-linked immunosorbent assays (ELISAs) for TGEV/PRCV differential serodiagnosis (17–20). Several commercial TGEV/PRCV blocking ELISAs are available, but comparative test performances have not been reported in recent publications. In this study, the diagnostic test performances of three commercial TGEV/PRCV blocking ELISA kits were evaluated using serum samples of precisely known porcine coronavirus immune status.

## RESULTS

### Clinical observations and virus shedding.

Mild watery diarrhea was observed in pigs inoculated with TGEV Miller strain between 2 to 4 days postinoculation (dpi). No clinical signs were observed in pigs from the negative-control, TGEV Purdue, and PRCV-inoculated groups throughout the experiment. All animals survived until the end of the study (42 dpi).

**FIG 1:**
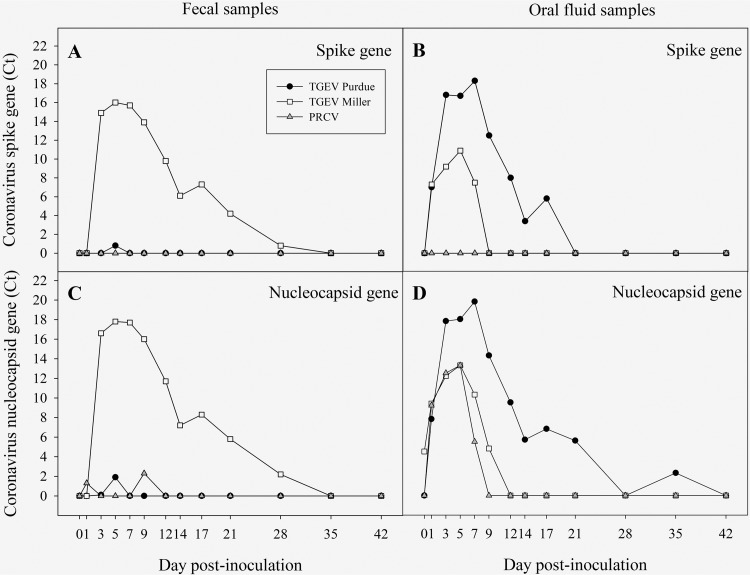
Detection of transmissible gastroenteritis virus (TGEV) and porcine respiratory coronavirus (PRCV) in pen-based feces and oral fluid samples by TGEV spike (S) and TGEV/PRCV nucleocapsid (N) gene-specific rRT-PCRs, as follows: S gene-specific PCR results in feces samples (A), S gene-specific PCR results in oral fluid samples (B), N gene-specific PCR results in feces samples (C), and N gene-specific PCR results in oral fluid samples (D). Results presented as mean adjusted quantification cycle (*C_T_*) (35 – sample *C_T_*) of positive samples.

All fecal samples collected from pigs in the TGEV strain Purdue, TGEV strain Miller, PRCV-inoculated, and negative-control groups were tested by rRT-PCR and found to be TGEV and PRCV negative prior to the inoculations. Likewise, all oral fluid samples, with the exception of one false-positive (threshold cycle [*C_T_*], 35.5) result in the TGEV Purdue group, were negative before inoculation. The detection of TGEV (S and N genes) and PRCV (N gene) in pen-based feces and oral fluids by rRT-PCR is shown in Fig. 1. Transmissible gastroenteritis virus was detected in feces between 3 and 28 dpi by rRT-PCR in pigs inoculated with TGEV strain Miller (Fig. 1A and C). No significant fecal shedding was detected in pigs inoculated with TGEV strain Purdue or PRCV throughout the study (Fig. 1A and C). Viral shedding was specifically detected by rRT-PCR in oral fluid samples collected from pigs inoculated with TGEV strain Purdue (1 to 21 dpi), TGEV strain Miller (1 to 9 dpi), and PRCV (1 to 7 dpi) (Fig. 1B and D).

### TGEV antibody response over the course of the experimental inoculation.

Serum samples collected from all groups of pigs prior to inoculation were antibody negative for porcine coronaviruses (TGEV, PRCV, porcine epidemic diarrhea virus [PEDV], and porcine delta coronavirus [PDCoV]). All pigs in the negative-control group remained TGEV and PRCV seronegative throughout the monitoring period when tested with any of the three TGEV/PRCV differential blocking ELISA kits evaluated in this study (Swinecheck TGEV/PRCV Recombinant [Biovet], INgezim Corona Diferencial [Ingenasa], and Svanovir TGEV/PRCV-Ab [Svanova] assays).

**FIG 2:**
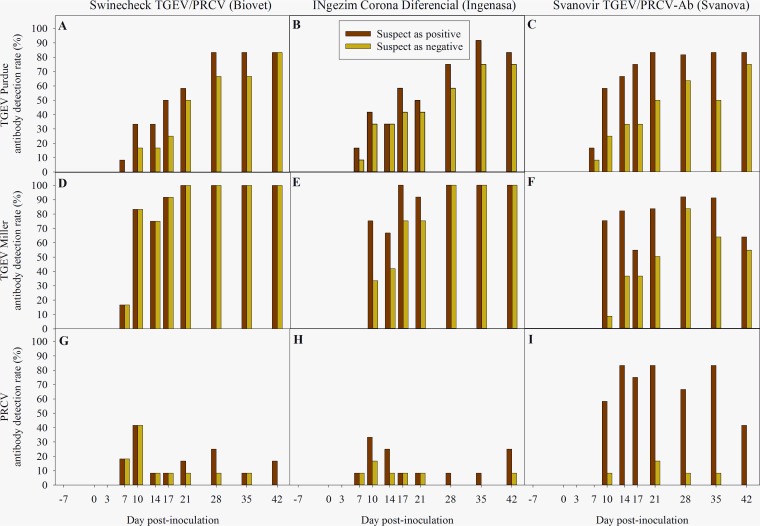
TGEV antibody detection rate (%) of the 3 commercial TGEV/PRCV blocking ELISA kits evaluated in this study, Swinecheck TGEV/PRCV Recombinant (Biovet, Canada) (A, D, and G), INgezim Corona Diferencial (Ingenasa, Spain) (B, E, and H), and Svanovir TGEV/PRCV-Ab (Svanova, Sweden) (C, F, and I). Results are presented considering suspect results to be positive (red bars) or negative (green bars).

The percentages of TGEV antibody-positive serum samples reported by the three commercial ELISA kits evaluated over the 50-day study period for pigs inoculated with TGEV strains Purdue and Miller are presented in Fig. 2A to F, respectively. Suspect results are presented as positives or negatives. The first TGEV-specific antibody detection was reported between 7 and 10 dpi in both TGEV-inoculated groups (strains Purdue and Miller). The number of TGEV antibody-positive pigs detected increased through the study, regardless of the ELISA kit used.

For the TGEV Purdue inoculation group, no significant differences (*P > *0.05) were found in the percentages of TGEV-seropositive pigs reported by the three ELISAs regardless of the time postinoculation and interpretation of suspect results. In contrast, for the TGEV Miller inoculation group, we found that a significantly higher (*P < *0.05) percentage of TGEV-seropositive animals was detected by the Swinecheck TGEV/PRCV Recombinant ELISA than by both the INgezim Corona Diferencial ELISA (dpi 10) and Svanovir TGEV/PRCV-Ab ELISA (10, 17, and 21 dpi) only when suspect results were interpreted as negative.

A nonspecific TGEV antibody response to PRCV-inoculated pigs was reported by the three ELISA kits between 7 and 42 dpi (Fig. 2G to I). No significant differences in the percentages of TGEV false positives were found between ELISAs over the monitoring period when TGEV suspect results were interpreted as negative (*P > *0.05). However, the overall proportion of false positives was higher when suspect results were interpreted as positive, regardless of the ELISA kit evaluated. Moreover, the percentage of TGEV-false-positive results reported by the Svanovir TGEV/PRCV-Ab ELISA was significantly greater (*P < *0.05) than those obtained with the Swinecheck TGEV/PRCV Recombinant ELISA (14, 21, and 35 dpi) and INgezim Corona Diferencial ELISA (21 to 35 dpi).

### PRCV antibody response over the course of the experimental inoculation.

With the exception of one false-positive result reported (42 dpi) by the INgezim Corona Diferencial ELISA kit, the negative-control group remained seronegative for PRCV by the three commercial ELISAs throughout the study.

**FIG 3:**
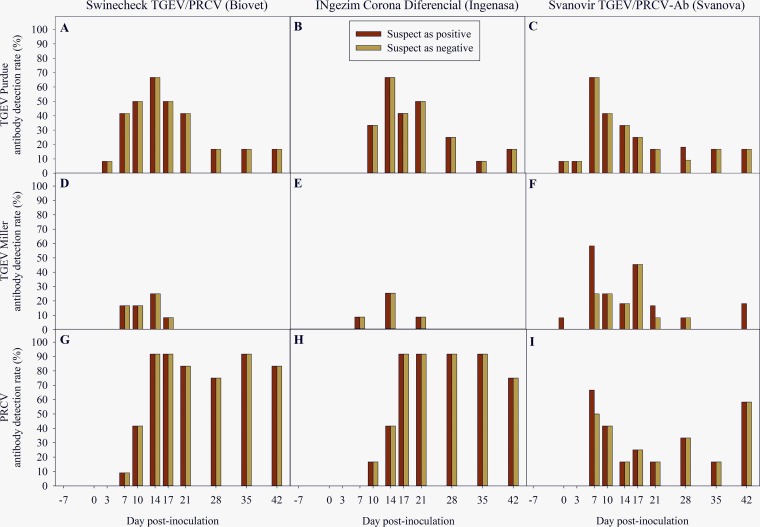
Porcine respiratory coronavirus (PRCV) antibody detection rate (%) of the 3 commercial TGEV/PRCV blocking ELISA kits evaluated in this study, Swinecheck TGEV/PRCV Recombinant (Biovet, Canada) (A, D, and G), INgezim Corona Diferencial (Ingenasa, Spain) (B, E, and H), and Svanovir TGEV/PRCV-Ab (Svanova, Sweden) (C, F, and I). Results are presented considering suspect results to be positive (red bars) or negative (green bars).

Figure 3 shows the percentage of PRCV serum samples detected by the three commercial ELISAs in PRCV-inoculated (specific detection; Fig. 3G to I) and TGEV-inoculated (nonspecific detection; Fig. 3A to F) animals over the course of the study.

On PRCV-inoculated animals, an early PRCV-specific antibody response was first detected at 7 to 10 dpi by the three commercial ELISAs and lasted through 42 dpi (Fig. 3G to I). An analysis of the proportion of PRCV-seropositive animals showed significant differences between ELISA kits over time following PRCV inoculation, regardless of the interpretation of suspect results (*P < *0.05). The percentage of PRCV-seropositive animals detected by the Svanovir TGEV/PRCV-Ab ELISA was significantly lower than those with both the Swinecheck TGEV/PRCV Recombinant ELISA (dpi 14 to 35) and INgezim Corona Diferencial ELISA (dpi 17 to 21 and 28) (*P < *0.05). No differences were found between the Swinecheck TGEV/PRCV Recombinant and INgezim Corona Diferencial ELISAs.

On TGEV-inoculated animals, a nonspecific PRCV serum antibody response was detected by the three commercial ELISAs. This serologic cross-reactivity seemed to be TGEV strain dependent, with a higher percentage of PRCV-false-positive results in the TGEV strain Purdue-inoculated group (Fig. 3A to C) than in the TGEV strain Miller group (Fig. 3D to F). The cross-reactivity appeared more marked at early stages postexposure (7 to 14 dpi). No differences were found between ELISA kits over the course of the study, except at 7 dpi when the proportion of PRCV-false-positive animals detected by the Svanovir TGEV/PRCV-Ab ELISA was significantly greater (*P < *0.05) than that by the INgezim Corona Diferencial ELISA regardless of the interpretation of suspect results.

### Comparative diagnostic performance of the TGEV/PRCV differential ELISAs.

The analytical specificity and diagnostic sensitivity and specificity of the three commercial differential TGEV/PRCV ELISA kits evaluated in this study are presented in Table 1. Diagnostic parameters are presented relative to the interpretation of suspect results as positive or negative.

**TABLE 1:** Estimated diagnostic sensitivity, diagnostic specificity, and analytical specificity of the 3 commercial TGEV/PRCV blocking ELISA kits evaluated in this study

Parameter	Group	% (95 confidence interval [%]) by assay and suspect result categorization					
		Suspect results as positive			Suspect results as negative		
		Swinecheck	INgezim	Svanovir	Swinecheck	INgezim	Svanovir
Diagnostic sensitivity^*a*^	TGEV Purdue	65 (63, 95)	65 (49, 81)	79 (61, 90)	51 (36, 70)	54 (38, 72)	50 (33, 67)
	TGEV Miller	94 (81, 99)	93 (81, 99)	78 (60, 89)	94 (81, 99)	82 (67, 94)	54 (38, 72)
	TGEV^*d*^	80 (64, 92)	79 (64, 92)	78 (60, 89)	73 (55, 86)	68 (64, 92)	53 (36, 70)
	PRCV	86 (71, 95)	85 (71, 95)	28 (14, 15)	86 (71, 95)	81 (64, 92)	28 (14, 15)
Diagnostic specificity^*b*^	TGEV	100 (90, 100)	100 (90, 100)	100 (90, 100)	100 (90, 100)	100 (90, 100)	100 (90, 100)
	PRCV	100 (90, 100)	99 (90, 100)	100 (90, 100)	100 (90, 100)	99 (90, 100)	100 (90, 100)
Analytical specificity^*c*^	TGEV Purdue	63 (46, 79)	70 (52, 83)	71 (52, 84)	63 (46, 79)	70 (52, 84)	72 (55, 86)
	TGEV Miller	91 (78, 98)	95 (81, 99)	76 (58, 88)	92 (78, 98)	95 (81, 99)	84 (67, 94)
	TGEV^*d*^	77 (61, 90)	82 (67, 94)	73 (55, 86)	77 (61, 90)	82 (67, 94)	78 (61, 90)
	PRCV	82 (67, 94)	84 (67, 94)	39 (23, 57)	87 (71, 95)	93 (78, 98)	95 (81, 99)

a The diagnostic sensitivity of each kit was calculated according to diagnosed and true statuses of each group on dpi 14 to 42 (considered positive status).

b The diagnostic specificity of each test was calculated according to diagnosed TGEV or PRCV status in the negative-control group on dpi −7 and 42.

c The analytical specificity of each test was calculated according to TGEV or PRCV status across groups between dpi 7 and 42.

d Combined results from pigs inoculated with both TGEV Purdue and Miller strains.

Overall, the diagnostic sensitivity for the TGEV Miller inoculation group was higher than for the TGEV Purdue group, independent of the commercial ELISA kit evaluated. The Swinecheck TGEV/PRCV Recombinant ELISA kit showed the highest diagnostic sensitivity for antibody detection in pigs inoculated with TGEV strain Miller (94%), regardless of the interpretation of suspect results. The diagnostic sensitivity for the TGEV strain Purdue-inoculated group was higher with the Svanovir TGEV/PRCV-Ab ELISA kit (79%) when suspect results were considered positive, while it was slightly higher with the INgezim Corona Diferencial ELISA kit (54%) when suspect results were interpreted as negative.

The diagnostic sensitivities for PRCV antibody detection obtained with both Swinecheck TGEV/PRCV Recombinant ELISA (86%) and INgezim Corona Diferencial ELISA (81 to 85%) were greater than that of the Svanovir TGEV/PRCV-Ab ELISA kit (28%). With the exception of the INgezim Corona Diferencial ELISA, the diagnostic sensitivity calculated for each ELISA kit was not affected by the interpretation of suspect results (positive versus negative).

The three commercial ELISAs showed 100% diagnostic specificity for both TGEV and PRCV detection, with the exception for one PRCV-false-positive result reported by the INgezim Corona Diferencial ELISA, which resulted in a diagnostic specificity of 99.2% for PRCV detection. The diagnostic specificity of any of the evaluated ELISA kits was unaffected by the interpretation of suspect results.

The INgezim Corona Diferencial ELISA kit showed the highest analytical specificity (less cross-reactivity) for TGEV-specific antibody detection (82.3%). For all three commercial ELISAs, we found higher cross-reactivity to PRCV within the TGEV Purdue inoculation group than in the TGEV Miller group. The highest analytical specificity for PRCV was obtained with the INgezim Corona Diferencial ELISA (84%) when suspect results were interpreted as positive or with the Svanovir TGEV/PRCV-Ab ELISA kit (95%) when suspect results were interpreted as negative; however, the Svanovir TGEV/PRCV-Ab ELISA kit showed the lowest analytical specificity (39%) of the kits when suspect results were interpreted as positive.

## DISCUSSION

The greatest risk of introducing TGEV is through the importation of pigs from endemically infected countries. Seronegative pigs of all ages are susceptible to infection with TGEV (1, 21, 22), with the route of infection typically being oral or oral-nasal (23, 24). To prevent TGEV from entering a naive herd, it is important to introduce only animals from TGEV-free and serologically negative herds, along with disciplined biosecurity. Therefore, quarantine and testing for TGEV-seronegative animals are requirements for export/import into a herd. Moreover, the detection of TGEV antibodies, particularly in reproductive animals, can assist in diagnosis and control of the disease. However, TGEV serology is complicated by cross-reactivity with PRCV (5), an S protein deletion mutant of TGEV with altered tissue tropism (nonenteropathogenic but respiratory) that circulates subclinically in most swine herds worldwide (7, 9, 25). This cross-reactivity may explain why a region endemic for PRCV has less TGEV-associated disease (26). Porcine respiratory coronavirus does not cause significant losses in swine except for its contribution to the porcine respiratory disease complex. Historically, PRCV diagnosis was based on recognition of respiratory disease in growing pigs that have high antibody titers to TGEV but have had no clinical enteric disease or lesions of atrophic enteritis. Conventional serological tests, such as virus neutralization, indirect fluorescent antibody, agar-gel precipitation, indirect immunoperoxidase, radioimmunoprecipitation, and some ELISAs based on polyclonal TGEV antibodies, cannot be used for differentiation between TGEV and PRCV, because the two viruses share antigenic determinants located on the spike (S), membrane (M), nucleoprotein (N), and envelope (E) structural proteins (27, 28). The exception is a large deletion (227 amino acids in length) in the amino terminus of the PRCV S protein that is responsible for the loss of hemagglutination activity (29). This deletion provided the opportunity to develop blocking enzyme-linked immunosorbent assays for differentiation of PRCV and TGEV infections and laid the basis for the commercial TGEV/PRCV differential ELISAs that are currently available (17–20). Few field reports have described the use of TGEV/PRCV blocking ELISAs to differentiate antibodies to TGEV and PRCV and their utility at the herd level (26, 30, 31). However, no previous reports have compared the diagnostic performances of different commercially available TGEV/PRCV blocking ELISAs. In this study, we assessed and compared the diagnostic performances (sensitivity, specificity, and cross-reactivity) of three commercial differential TGEV/PRCV blocking ELISA kits (i.e., Swinecheck TGEV/PRCV Recombinant ELISA [Biovet, Canada]; INgezim Corona Diferencial ELISA [Ingenasa, Spain]; and Svanovir TGEV/PRCV-Ab ELISA kit [Svanova, Sweden]) at the individual pig level using experimental samples of precisely known porcine coronavirus immune status. The use of samples with the same disease/immune status allowed for accurate interpretation of test results, including those that were discordant or unexpected.

Molecular diagnostic methods (e.g., *in situ* hybridization and multiplex rRT-PCR) targeting both the conserved and PRCV deletion regions are utilized to differentiate TGEV and PRCV in infected pigs (8, 32, 33). Specimens primarily used for TGEV virus detection include feces and intestinal contents, and those for PRCV detection include nasal swabs or lung homogenates. In this study, the verification and monitoring of the TGEV or PRCV viral shedding status were performed using rRT-PCR testing of pen-based feces and oral fluids. This process provided further information on the dynamics of viral shedding in feces versus oral fluids. Viral shedding was detected in both feces and oral fluids. However, for pigs inoculated with PRCV and, interestingly, for TGEV Purdue, virus shedding was only detected in oral fluids. This could be related to differences in pathogenicity and tissue tropism, which were beyond the scope of this study. These results indicated that oral fluids may replace feces as a more suitable specimen for direct detection and surveillance of TGEV/PRCV by rRT-PCR.

Overall, we observed differences in the test performances of the three commercial ELISA kits evaluated in this study. However, the differences were more marked for the Svanovir TGEV/PRCV-Ab ELISA kit, compared to either the Swinecheck TGEV/PRCV Recombinant ELISA or the INgezim Corona Diferencial ELISA, for which test performance was comparable. This could be due to differences/similarities in assay design. For instance, both the Swinecheck TGEV/PRCV Recombinant ELISA and the INgezim Corona Diferencial ELISA use a TGEV recombinant S protein antigen on a plate, while the Svanovir TGEV/PRCV-Ab ELISA claims to use noninfectious TGEV antigen of an unknown source, which could detect antibodies to a variety of viral proteins. The way the antigen is presented may also affect assay performance. The antigen can either be bound directly to the plate wells (as in the Swinecheck TGEV/PRCV Recombinant ELISA and Svanovir TGEV/PRCV-Ab ELISA) or may be captured by antigen-specific antibodies immobilized on the plate (as in the INgezim Corona Diferencial ELISA). In addition, the Svanovir TGEV/PRCV-Ab ELISA is the only kit using unlabeled TGEV and TGEV/PRCV mouse monoclonal antibodies (MAbs), which requires an additional incubation step with anti-mouse horseradish peroxidase (HRP)-conjugated secondary antibody. Undisclosed differences in buffer composition could also be involved in the differences in assay performance among the kits.

All three ELISA kits showed higher diagnostic sensitivity in the detection of anti-TGEV antibodies in pigs inoculated with TGEV strain Miller (77.9 to 94.4%) than in pigs inoculated with TGEV strain Purdue (65.3 to 78.9%), regardless of the kit used. The differences in diagnostic sensitivity among TGEV-inoculated groups could be due to strain-related differences in virulence, which may have impacted the magnitude of the immune response. Indeed, in this study, pigs inoculated with TGEV strain Miller showed mild watery diarrhea between 2 and 4 dpi, while no clinical signs or viral shedding in feces were observed in the pigs inoculated with TGEV Purdue.

The diagnostic specificity of the three commercial ELISA kits was evaluated on pigs of precisely known negative porcine coronavirus immune status (i.e., the porcine coronavirus negative-control group) to rule out potential cross-reactivity with other porcine coronaviruses (i.e., pigs virologically and serologically negative for PEDV, PDCoV, porcine hemagglutinating encephalomyelitis virus [PHEV], TGEV, and PRCV). All three kits evaluated in this study showed excellent diagnostic specificity, ranging from 99% (Svanovir TGEV/PRCV-Ab ELISA) to 100% (Swinecheck TGEV/PRCV Recombinant ELISA and INgezim Corona Diferencial ELISA). These results were consistent with previous reports in the field where a TGEV/PRCV blocking ELISA (i.e., Swinecheck and Biovet) showed a diagnostic specificity of 100% in wild boar populations historically free from coronavirus disease (34). With the final goal of maximizing both diagnostic sensitivity and specificity (but understanding that there is a balance between the two parameters), diagnostic specificity is of paramount importance during TGEV/PRCV screening due to the impact of false-positive results on global pig trade operations. The false-positive rate of any diagnostic test is a function of the specificity of the test and the prevalence of the disease.

The assessment of the selectivity of the antigen-antibody response (analytical specificity) was conducted on each of the three commercial blocking ELISAs using a panel of heterologous monotypic known-status-positive sera generated under experimental conditions. Specifically, in this study, the analysis of the analytical specificity was circumscribed to the cross-reactivity between TGEV (strains Purdue and Miller) and PRCV. However, the potential cross-reactivity between TGEV/PRCV against other swine coronaviruses has previously been reported (35). A test was considered analytically specific for TGEV or PRCV when it did not react against heterologous positive sera. As with the diagnostic sensitivity, the overall analytical specificity varied among and across kits and groups of inoculation, even with intrakit variations depending upon the interpretation of suspect results. Serological cross-reactivity between TGEV and PRCV was previously reported by use of an immunoblotting assay based on TGEV/PRCV structural proteins (S, M, and N) and polyclonal antisera (11). This study further confirms that the serological cross-reactivity between TGEV and PRCV is significant even when using TGEV/PRCV differential blocking ELISAs. Overall, in this study, we observed a poor analytical specificity (i.e., high cross-reactivity) for PRCV antibody detection in pigs exposed to TGEV strain Purdue (63 to 72%), with no significant differences among kits. However, with the exception of the Svanovir TGEV/PRCV-Ab ELISA (76 to 84%), we found an acceptable analytical specificity for PRCV antibody detection in pigs exposed to TGEV Miller using the Swinecheck TGEV/PRCV Recombinant ELISA (91 to 92%) and the INgezim Corona Diferencial ELISA (95%). Current TGEV/PRCV differential immunoassays are based on a blocking ELISA format, which is inherently more specific than indirect ELISAs. In the blocking ELISA format, the degree to which specific antibodies in the test serum sample prevent binding of an agent-specific MAb is measured. Therefore, the higher the levels of specific antibodies are in a serum sample, the lower the likelihood for cross-reactivity. In fact, the overall higher TGEV antibody detection rate observed within the group inoculated with TGEV strain Miller correlates with the lower cross-reactivity against PRCV. That would also explain the overall higher rate of serologic cross-reactivity (regardless of the ELISA kit used) reported during the first few weeks postinoculation, when specific antibody levels are still low.

Previous reports indicated that the accuracy of the commercial TGEV/PRCV blocking ELISAs for differentiating between U.S. TGEV and PRCV strains was low (14, 15, 25). Individual test results must be interpreted with caution, particularly in the event of “suspect” results. We have demonstrated that interpretation of suspect results can impact significantly the diagnostic performance of any of the commercial kits evaluated in this study, with differences in their robustness in response to changes in the interpretation of suspect results. While analytical specificity improved overall when suspect results were interpreted as negative, this often comes at the cost of diagnostic sensitivity. In the event of suspect, undetermined, or unexpected positive results, it is recommended that serology testing on paired serum samples be conducted, in which a second sample should be drawn 10 to 14 days after the first sample collection. Paired serum samples should be tested together to maximize the diagnostic value of test results. Moreover, false-positive results reported at early stages postexposure (when animals are actively shedding virus) could be confirmed by rRT-PCR on oral fluid specimens, as evidenced in this study.

The presence of TGEV remains a barrier to international livestock trade (36). The blocking ELISA format alone was determined to be useful in large swine populations for the detection of TGEV-infected herds. Moreover, the ability to specifically detect PRCV antibodies minimized the probability of false-positive TGEV results and subsequent exclusion of those herds for trading. However, this study demonstrates that the serologic cross-reactivity between TGEV and PRCV at the individual pig level affects the accuracy of commercial blocking ELISAs. Therefore, it is important to remember that the TGEV/PRCV blocking ELISAs should only be applied on a herd basis (14, 15, 17, 19).

## MATERIALS AND METHODS

### Experimental design.

“Known-status” serum samples (*n* = 528) collected from pigs experimentally inoculated with TGEV Purdue (*n* = 12), TGEV Miller (*n* = 12), PRCV (*n* = 12), or with culture medium (minimum essential medium [MEM] Life Technologies, Carlsbad, CA) (negative control; *n* = 12) were used to evaluate the diagnostic performance (diagnostic sensitivity and specificity) and antibody cross-reactivity (analytical specificity) of the following three commercial TGEV/PRCV blocking ELISAs: (i) Swinecheck TGEV/PRCV Recombinant (Biovet, Canada), (ii) INgezim Corona Diferencial (Ingenasa, Spain), and (iii) Svanovir TGEV/PRCV-Ab (Svanova, Sweden). Commercial ELISAs were performed and the test results interpreted according to the manufacturer’s instructions.

### Viral inoculum.

Swine testicle (ST) cell culture adapted to TGEV Purdue (ATCC VR-763), TGEV Miller (ATCC VR-1740), and PRCV (ATCC VR-2384) were obtained from the American Type Culture Collection (ATCC, Manassas, VA). In brief, ST cells (ATCC CRL-1746) were cultured in 25-cm^2^ flasks (Corning, Corning, NY) using MEM (Life Technologies) supplemented with 10% fetal bovine serum (Life Technologies), 2 mM l-glutamine (Sigma-Aldrich, St. Louis, MO), 0.05 mg/ml gentamicin (Life Technologies), 10 units/ml penicillin (Life Technologies), 10 µg/ml streptomycin (Sigma-Aldrich), and 0.25 µg/ml amphotericin (Sigma-Aldrich). At 100% confluence of the cell monolayer, the maintenance medium was decanted, and the monolayer was washed twice with maintenance medium. Thereafter, each flask of cells was inoculated with 500 µl of each virus mixed with postinoculation medium, i.e., MEM supplemented with 0.3% tryptose phosphate broth (Sigma-Aldrich) and 0.02% yeast extract (Sigma-Aldrich). The cells were incubated at 37°C with 5% CO_2_ for 2 h to allow virus adsorption. After incubation, 5 ml of postinoculation medium was added to each flask without removing viral inoculum. The flasks were incubated at 37°C with 5% CO_2_ and subjected to one freeze-thaw cycle at −80°C when 80% cytopathic effect (CPE) was observed. Cell debris was removed by centrifugation at 3,000 × *g* for 10 min at 4°C, and the virus content was harvested by collecting the supernatant. Virus titration was performed on confluent ST cell monolayers grown in 96-well plates (Costar; Corning) using a method described elsewhere (37).

### Animals.

The animal study was conducted at the Iowa State University (ISU) Livestock Infection Disease Isolation Facility (LIDIF) with the approval of the Iowa State University Office for Responsible Research. Forty-eight 7-week-old conventional pigs were acquired from a commercial wean-to-finish farm with no history of porcine coronavirus infection. During the herd prescreening process and prior to the beginning of the study, fecal and nasal swabs from all animals were tested for TGEV, PRCV, porcine hemagglutinating encephalomyelitis virus (PHEV), porcine epidemic diarrhea virus (PEDV), and porcine delta coronavirus (PDCoV) by real-time reverse transcription-PCR (rRT-PCR) assays. Serum samples were tested for all of the pathogens listed above using antibody-based methods, as described elsewhere (38–40).

Upon arrival, all animals were ear-tagged and randomly assigned to one of four inoculation groups (*n* = 12 per group in the TGEV Miller, TGEV Purdue, PRCV, and negative-control groups). Pigs within each group were housed in pens of 2 pigs in the same room. Each pen was equipped with nipple drinkers, and pigs were fed twice daily with an antibiotic-free commercial diet (Heartland Co-op, West Des Moines, IA, USA). Details regarding viral dose and route of inoculation for each group are presented in Table 2. The infectious dose used for each virus was previously determined in a pilot study (data not shown) with the objective of stimulating a humoral response effectively. Pigs were evaluated clinically twice daily throughout the study. The TGEV and PRCV infectious status of every pig was established and monitored throughout the study by rRT-PCR tests on fecal and oral fluid samples and ELISA-based tests on serum samples.

**TABLE 2:** TGEV and PRCV strains used, inoculum doses, and routes of inoculation

Inoculation group	Strain	GenBank accession no.	Virus propagation		Inocula (per pig)			
			Cell line	Virus passage no.	Virus titer (TCID_50_/ml)^*a*^	Virus culture vol (ml)	Total vol (ml)^*b*^	Inoculation route
TGEV Miller	ATCC VR-1740	DQ811785	Swine testicle (ATCC CRL-1746)	16	4.0 × 10^6^	35	40	Orogastric
TGEV Purdue	ATCC VR-763	DQ811789	Swine testicle (ATCC CRL-1746)	12	2.4 × 10^8^	30	35	Orogastric
PRCV	ATCC VR-2384	DQ811787	Swine testicle (ATCC CRL-1746)	18	4.0 × 10^5^	15	20	Nasal (10 ml per nostril)
Sham (culture medium)							20	Oronasal

a TCID_50_, 50% tissue culture infectious dose.

b TGEV strains were mixed with milk replacer (Esbilac; PetAg, Inc., Hampshire, IL) or culture medium (PRCV).

### Sample collection.

Blood samples were collected on −7, 0, 3, 7, 10, 14, 17, 21, 28, 35, and 42 days postinoculation (dpi) from the jugular vein or cranial vena cava using a single-use blood collection system (Becton Dickinson, Franklin Lakes, NJ) and serum separation tubes (Kendall, Mansfield, MA). Serum was separated by centrifugation at 1,500 × *g* for 5 min, aliquoted into 2-ml cryogenic tubes (Greiner Bio-One GmbH, Frickenhausen, Germany), and stored at −80°C until use.

Floor fecal samples were collected daily from each pen (2 pigs per pen) within each inoculation group (6 pens per group) from −7 to 42 dpi. Approximately 2 ml of feces was placed in a 2-ml cryogenic tube (BD Falcon). Fecal samples (100 µl) from each group on the same day postinoculation were pooled into a 2-ml cryogenic tube (BD Falcon) at the end of the study for rRT-PCR testing.

Oral fluid samples were collected from each pen within each inoculation group twice a day (7:00 a.m. and 1:00 p.m.) from −7 to 42 dpi. In brief, 3-strand 1.6-cm 100% cotton rope (Web Rigging Supply, Inc., Carrollton, GA) was hung from a bracket fixed to one side of each pen for 30 min, during which time the pigs chewed on and interacted with the rope. After 30 min, the wet end of the rope was severed, sealed in a plastic bag, and then passed through a clothes wringer (Dyna-Jet, Overland Park, KS). The oral fluid accumulated in the bottom of the bag was decanted into 50-ml conical tubes (Corning), aliquoted into 2-ml cryogenic tubes (Greiner Bio-One GmbH), and stored at −80°C.

### Real-time reverse transcription-PCR.

Feces and oral fluid samples were submitted to the ISU Veterinary Diagnostic Library (VDL) for porcine coronavirus screening by rRT-PCR. Transmissible gastroenteritis virus-specific spike (S) and TGEV/PRCV-specific nucleocapsid (N) genes were targeted and amplified by dually labeled probe-based rRT-PCR. In brief, sample RNA was extracted and eluted using the Ambion MagMAX viral RNA isolation kit (Life Technologies) and a KingFisher 96 magnetic particle processor (Thermo Fisher Scientific) following the procedures provided by the manufacturers. The TGEV S gene and the TGEV/PRCV N gene-based rRT-PCR were modified from previous procedures (40) and performed routinely at the ISU-VDL using the TaqMan Fast 1-step mastermix (Thermo Fisher Scientific). The RT-PCRs were conducted on an ABI 7500 Fast instrument (Life Technologies) as follows: 50°C for 5 min, 95°C for 20 s, 95°C for 3 s (40 cycles), and 60°C for 30 s. The rRT-PCR results were analyzed using an automatic baseline, with a threshold value of 0.1. A quantification cycle (*C_T_*) value of <35 was considered positive for both S and N gene-based rRT-PCR. Samples were considered positive for TGEV when both S and N genes were rRT-PCR positive. For PRCV, samples were considered positive when the S gene rRT-PCR was negative and the N gene rRT-PCR was positive. Transmissible gastroenteritis virus and PRCV *C_T_* data were reported as “adjusted *C_T_*,” calculated as shown in the following equation:

$$\text{Adjusted}\;C_{T}=\left(\text{cutoff}\;C_{T}\;\text{value}-\text{sample}\;C_{T}\;\text{value}\right)$$

### TGEV/PRCV differential blocking ELISAs.

Serum samples were tested for TGEV- and PRCV-specific antibodies using the following three commercially available TGEV/PRCV differential blocking ELISA kits: (i) Swinecheck TGEV/PRCV Recombinant (Biovet, Canada), (ii) INgezim Corona Diferencial (Ingenasa, Spain), and (iii) Svanovir TGEV/PRCV-Ab (Svanova, Sweden). All assays were performed according to the manufacturers’ instructions. The pig serum samples and controls were first added to the TGEV antigen-coated wells for the first incubation step. In the INgezim Corona Diferencial ELISA, plate wells are coated with a recombinant TGEV S protein which is captured by a specific monoclonal antibody (MAb). The Swinecheck TGEV/PRCV Recombinant ELISA is also based on a recombinant TGEV S protein but directly coated on plate wells, while the Svanovir TGEV/PRCV-Ab ELISA uses noninfectious TGEV antigen (source unknown) as a coating antigen. If anti-TGEV or anti-PRCV antibodies are present in the test sample, they will bind to the viral (TGEV) antigen in the wells and block the antigenic sites. In contrast, if anti-TGEV or anti-PRCV antibodies are absent in the test sample, these sites will remain free. Then, after a washing step to eliminate unbound antibodies, a horseradish peroxidase (HRP)-conjugated mouse anti-TGEV MAb targeting the N-terminal region of the S glycoprotein that is deleted in PRCV, or an anti-TGEV/PRCV MAb that binds to conserved regions of both TGEV and PRCV, is added into the first well (odd column) or second well (even column), respectively, and attaches to specific free sites of the virus. Thus, when antibodies in the test sample occupy the binding sites on the antigen, the binding of the conjugated MAbs is blocked. If anti-TGEV or anti-PRCV antibodies are absent in the test sample, these sites will remain free. The amount of antibody in the test sample that is bound to the TGEV antigen is inversely proportional to the intensity of the color. Antibody ELISA results were expressed as the percentage of inhibition (Swinecheck TGEV/PRCV and Svanovir TGEV/PRCV-Ab) or optical density (INgezim Corona Diferencial) and interpreted (positive, negative, or suspect/inconclusive) for TGEV and PRCV according to the manufacturers’ instructions. The diagnostic sensitivity, diagnostic specificity, and analytical specificity of the three commercial ELISAs were calculated based on the true status of the samples compared to the specific detection of TGEV (Miller and/or Purdue) and/or PRCV antibodies. Specifically, porcine coronavirus-negative serum samples (*n* = 204) were used to estimate diagnostic specificity for the three ELISA kits. Serum samples positive for TGEV (Purdue and Miller strains) (*n* = 144) collected between 14 and 42 dpi and PRCV-positive samples (*n* = 72) collected between 14 and 42 dpi were used to calculate the time of detection, antibody detection over time, and the overall diagnostic sensitivity of the three ELISA kits for TGEV and PRCV Ab detection, respectively.

### Data analysis.

Analyses were conducted using commercial statistical software (SAS version 6.1.7601; Microsoft Corporation, USA). The percentage of results for TGEV/PRCV ELISA were interpreted (positive, negative, or suspect) according to the manufacturer’s recommendations. The percentage of seropositive animals per day postinoculation detected by each kit were analyzed in two ways, by considering suspect results to be positive or by considering suspect results to be negative. Significant differences (*P < *0.05) in ELISA-positive rates relative to TGEV (Purdue or Miller strains) and PRCV were calculated using Pearson’s chi-square test or Fisher’s exact test. The diagnostic sensitivity and specificity of the three commercial ELISAs were evaluated based on experimental serum samples of precisely known porcine coronavirus immune status (i.e., samples collected on <7 dpi were negative, and serum samples collected on >7 dpi were positive). Analytical specificity (bidirectional cross-reactivity) between TGEV versus PRCV was evaluated using samples (*n* = 288) collected between 7 and 42 dpi from animals inoculated with TGEV strain Purdue (*n* = 96), TGEV strain Miller (*n* = 96), and PRCV (*n* = 96) in each given case. Figures were created using commercial graphics software (SigmaPlot version 12.5; Systat Software, Inc.).
